# Association between HIF-1α C1772T/G1790A polymorphisms and cancer susceptibility: an updated systematic review and meta-analysis based on 40 case-control studies

**DOI:** 10.1186/1471-2407-14-950

**Published:** 2014-12-15

**Authors:** Qing Yan, Pin Chen, Songtao Wang, Ning Liu, Peng Zhao, Aihua Gu

**Affiliations:** Department of Neurosurgery, The First Affiliated Hospital, Nanjing Medical University, 300 Guangzhou Road, Nanjing, 210029 China

**Keywords:** HIF-1 gene, Polymorphism, Cancer, Susceptibility, Meta-analysis

## Abstract

**Background:**

HIF-1 (hypoxia-inducible factor 1) is a transcriptional activator that functions as a critical regulator of oxygen homeostasis. Recently, a large number of epidemiological studies have investigated the relationship between HIF-1α C1772T/G1790A polymorphisms and cancer susceptibility. However, the results remain inconclusive. Therefore, we performed a meta-analysis on all of the available case-control studies to systematically summarize the possible association.

**Methods:**

A literature search was performed using PubMed and the Web of Science database to obtain relevant published studies. Pooled odds ratios (ORs) and corresponding 95% confidence intervals (CIs) for the relationship between HIF-1α C1772T/G1790A polymorphisms and cancer susceptibility were calculated using fixed- and random-effects models when appropriate. Heterogeneity tests, sensitivity analyses and publication bias assessments were also performed in our meta-analysis.

**Results:**

A total of 40 studies met the inclusion criteria were included in the meta-analysis: 40 studies comprised of 10869 cases and 14289 controls for the HIF-1α C1772T polymorphism and 30 studies comprised of 7117 cases and 10442 controls for the HIF-1α G1790A polymorphism. The results demonstrated that there were significant association between the HIF-1α C1772T polymorphism and cancer susceptibility under four genetic models (TT *vs.* CC: OR = 1.63, 95% CI = 1.02-2.60; CT + TT *vs.* CC: OR = 1.15, 95% CI = 1.01-1.34; TT *vs.* CT + CC: OR = 2.11, 95% CI = 1.32-3.77; T *vs.* C: OR = 1.21, 95% CI = 1.04-1.41). Similarly, the statistically significant association between the HIF-1α G1790A polymorphism and cancer susceptibility was found to be consistently strong in all of the genetic models. Moreover, increased cancer risk was observed when the data were stratified by cancer type, ethnicity and the source of controls.

**Conclusions:**

This meta-analysis demonstrates that both the C1772T and G1790A polymorphisms in the HIF-1α gene likely contribute to increased cancer susceptibility, especially in the Asian population and in breast cancer, lung cancer, pancreatic cancer and oral cancer. However, further research is necessary to evaluate the relationship between these polymorphisms and cancer risk.

**Electronic supplementary material:**

The online version of this article (doi:10.1186/1471-2407-14-950) contains supplementary material, which is available to authorized users.

## Background

Human cancer is a major cause of death in the world, and it is estimated that the number of new cases will increase to more than 15 million in the coming decade, creating a substantial worldwide public health burden [1, 2]. Various factors, such as genetic and environmental influences, are associated with cancer prognosis. However, the exact etiology and mechanism of carcinogenesis have not yet been clearly elucidated. In recent years, it has become well-accepted that intrinsic factors, such as host genetic susceptibility, may play important roles in the process of cancer development [3, 4], and an increasing number of studies have focused on the association between genetic factors and cancer susceptibility.

Hypoxia-inducible factor 1 (HIF-1) is a transcriptional activator that functions as a critical regulator of oxygen homeostasis. It is a heterodimer composed of two subunits, HIF-1α and HIF-1β, which dimerize and bind to DNA via the basic helix-loop-helix Per/Arnt/Sim (bHLH-PAS) domain [5, 6]. HIF-1α expression is induced in hypoxic cells, and its level exponentially increase when the cells are exposed to O_2_ concentration of less than 6%. Under hypoxic condition, HIF-1α ubiquitination decreases dramatically, resulting in an accumulation of the protein, while under normoxic condition, HIF-1α is rapidly degraded through von Hippel-Lindau (VHL)-mediated ubiquitination and proteasomal degradation [7–10]. HIF-1 has also been suggested to play an important role in tumor development, progression and metastasis, and HIF-1 can activate the transcription of more than 60 target genes that are involved in crucial aspects of cancer establishment, including cell survival, glucose metabolism, angiogenesis and invasion [11, 12].

The HIF-1α gene is located on chromosome 14q21-24, and recent studies have shown that there are a total of 35 common single nucleotide polymorphisms (SNPs) throughout the HIF-1α gene in Caucasian and Asian population [13–15]. Two important SNPs in exon 12 of the HIF-1 gene, HIF-1α C1772T (rs11549465) and HIF-1α G1790A (rs11549467), lead to amino acid substitution of proline to serine at position 582 and alanine to threonine at position 588 of the protein, respectively [8, 16, 17]. These two polymorphisms have been demonstrated to be functionally meaningful, resulting in increased transcriptional activity of HIF-1α [14, 18]. Previous studies have shown that the overexpression of HIF-1α is significantly associated with cell proliferation, increased tumor susceptibility, tumor size, lymph node metastasis and prognosis [19, 20].

In recent years, the HIF-1α gene has been a research focus in the scientific community, and many epidemiological studies have been performed to assess the association between HIF-1α C1772T/G1790A polymorphisms and cancer susceptibility. However, the results of the different studies are conflicting. Hence, we performed a meta-analysis of all of the eligible studies to clarify the role of HIF-1α C1772T/G1790A polymorphisms in cancer development.

## Methods

### Study eligibility and validity assessment

We performed a computerized literature search of the PubMed and Web of Science databases to identify all of the relevant studies of cancer that contained sufficient genotyping data for at least one of the two polymorphisms, HIF-1α C1772T or HIF-1α G1790A. The search strategy was designed by two researchers and included the following keywords: “HIF-1 OR hypoxia-inducible factor-1” and “polymorphism”, and the last search was updated on September 20th, 2013. To obtain all eligible publications, we also manually reviewed the references of the selected articles to identify other potential eligible publications. Articles investigating the association between cancer risk and the HIF-1α polymorphisms were identified with no language restriction.

### Inclusion criteria

The studies selected were required to meet the following criteria: 1) evaluate the association between the HIF-1α C1772T and/or HIF-1α G1790A polymorphisms and cancer risk; 2) use a human case-control design; 3) contain sufficient published data for the estimation of an odds ratio (OR) with a 95% confidence interval (CI).

### Data extraction

Data were extracted from all of the eligible publications by two investigators (Yan and Chen) independently, according to the inclusion criteria listed above. Disagreements between the two investigators were resolved by discussion until a consensus was reached. The following information was extracted from each of the included publications: the first author’s name, publication data, country of origin, ethnicities of the sample population (categorised as Asians, Caucasians and Mixed), cancer type, source of control group (population- or hospital-based controls), total number of cases and controls, and the number of cases and controls with the HIF-1α C1772T/G1790A polymorphisms.

### Statistical methods

The strength of the association between the HIF-1α C1772T/HIF-1α G1790A polymorphisms and cancer risk was measured by ORs with 95% CIs. The statistical significance of the pooled OR was calculated by the Z test, a *P* < 0.05 was considered to be statistically significant (*P*-values were two sided). For HIF-1α C1772T polymorphism, we examined the overall ORs and compared the cancer incidence using the allelic model (T versus C), homozygote model (TT versus CC), heterozygote model (TC versus CC), dominant model (TT + TC versus CC), recessive model (TT versus TC + CC). For HIF-1α G1790A polymorphism, we evaluated the risk in the allelic model (A versus G), homozygote model (AA versus GG), heterozygote comparison model (GA versus GG), dominant models (AA + AG versus GG), and recessive model (AA versus AG + GG). Subgroup analyses were also conducted by ethnicity, cancer type (“other cancer groups” means any cancer types with less than two separate publications) and source of controls. Statistical heterogeneity was estimated by a chi-square based Q-test, and when *P* < 0.05, the heterogeneity was considered to be significant. We combined all of the values from each individual study using the fixed-effect model and the random-effect model. When *P* > 0.05, the effects were assumed to be homogenous, and the fixed-effect model (the Mantel-Haenszel method) was used [21]. When *P* < 0.05, the random-effect model (the DerSimonian and Laird method) was more appropriate [22]. The inter-study variance I^2^ (I^2^ = 100% × (Q-df)/Q) was used to quantitatively estimate the heterogeneity, and the percentage of I^2^ was used to describe the extent of the heterogeneity, I^2^ < 25%, 25-75% and >75% represent low, moderate and high inconsistency, respectively [23, 24]. In addition, we performed sensitivity analyses to evaluate the potential biases of the results in our meta-analyses. The Hardy-Weinberg equilibrium (HWE) of the controls for each study was also calculated using a goodness-of-fit test (chi-square or Fisher’s exact test) and *P* < 0.05 was considered to be statistically significant. Sensitivity analyses were carried out to assess the stability of the results by conducting analysis of studies with controls in HWE. Finally, the Begg’s funnel plot and Egger’s test were utilised to estimate the publication bias [25]. All analyses were conducted by the software Stata (Version 11; Stata Corporation, College Station, Texas, USA). All *P*-values were two-sided and a *P* of < 0.05 was considered to be statistically significant.

## Results

### Studies selected

**Figure 1:**
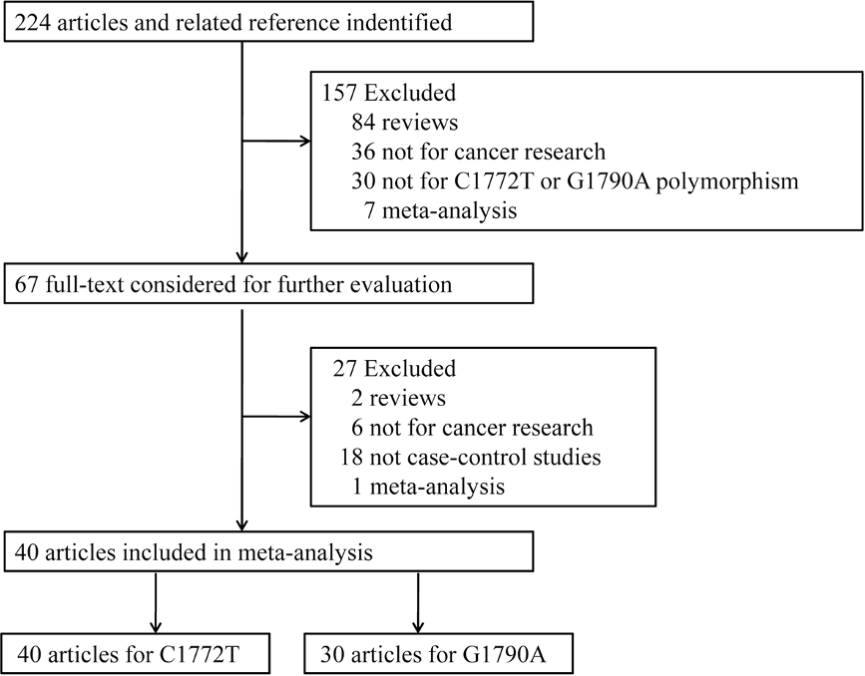
**Study flow-chart illustrating the literature search and eligible study selection process.**

Through the literature search and selection, a total of 40 eligible studies met the inclusion criteria and were included in our meta-analysis. One study (Konac *et al.*) [26] provided data on three types of cancer (cervical cancer, ovarian cancer, and endometrial cancer) and both polymorphisms; therefore, we have grouped them as one in the meta-analyses of all subjects except when stratified by cancer type. Thus, each type of cancer in this study was treated as a separated study in sub-group analyses. Among the 40 eligible studies, 40 studies, representing 10869 cases and 14289 controls, were ultimately analyzed for the HIF-1α C1772T polymorphism [8, 17, 26–63], and 30 studies, representing 7177 cases and 10442 controls, were analyzed for the HIF-1α G1790A polymorphism [8, 17, 26, 29–31, 33–35, 37–43, 45–48, 50, 52–57, 59, 62, 63]. The literature search and study selection procedure are shown in Figure 1. Of the 40 studies on the HIF-1α C1772T polymorphism, 6 studies were conducted on prostate cancer, 6 studies on breast cancer, 3 studies on lung cancer, 4 studies on colorectal cancer, 4 studies on renal cancer, 4 studies on oral cancer and 12 studies on other cancers. Among these eligible studies, 20 were studies on Asians, 16 were studies on Caucasians and 4 studies were performed on a population of mixed ethnicity. The control sources were population-based in 17 studies and hospital-based in 23 studies. For the HIF-1α G1790A polymorphism, 15 of the 30 eligible studies were performed in Asian populations, 13 studies were performed in Caucasian populations and 2 studies were performed in a mixed ethnicity population. Of these studies, 4 studies were conducted on breast cancer, 3 studies on lung cancer, 4 studies on oral cancer, 3 studies on prostate cancer, 3 studies on cervical cancer, 2 studies on pancreatic cancer, 2 studies on colorectal cancer, 4 studies on renal cancer and 7 studies on other cancers. The control sources were population-based in 17 studies and hospital-based in 13 studies. The genotype frequency data of the HIF-1α C1772T and HIF-1α G1790A polymorphisms were extracted from all of these eligible publications. For the HIF-1α C1772T polymorphism, the distributions of the genotypes in the control groups in 11 studies were not in HWE [17, 50, 51, 53, 54, 56–58, 60–62]. For the HIF-1α G1790A polymorphism there was 1 study not in HWE [62]. The main characteristics of the eligible studies in the meta-analysis are listed in Table 1.

**Table 1:** **Characteristics of studies included in the meta-analysis**

First author	Year	Country	Ethnicity	Cancer type	Gene type	Source of controls	Cases	Controls		Case		Control		HWE	
									MM	MW	WW	MM	MW	WW	
Clifford	2001	UK	Caucasian	Renal	C1772T	PB	48	143	42	6	0	110	27	6	0.02
					G1790A	PB	48	144	47	1	0	140	4	0	0.87
Tanimoto	2003	Japan	Asian	HNSCC	C1772T	PB	55	110	45	10	0	98	12	0	0.55
					G1790A	PB	55	110	51	4	0	101	9	0	0.65
Kuwai	2004	Japan	Asian	Colorectal	C1772T	PB	100	100	100	0	0	89	11	0	0.56
Ollerenshaw	2004	UK	Caucasian	Renal	C1772T	PB	160	162	16	54	90	1	90	71	0.001
					G1790A	PB	146	288	65	67	14	239	39	10	0.001
Ling	2005	China	Asian	Esophageal	C1772T	HB	95	104	84	11	0	93	11	0	0.57
Chau	2005	USA	Caucasian	Prostate	C1772T	PB	196	196	161	29	6	179	14	3	0.002
Fransen	2006	Sweden	Caucasian	Colorectal	C1772T	PB	198	258	167	28	3	213	43	2	0.92
Fransen	2006	Sweden	Caucasian	Colorectal	G1790A	PB	198	256	189	9	0	247	9	0	0.77
Konac	2007	Turkey	Caucasian	Cervical	C1772T	HB	32	107	10	14	8	68	37	2	0.23
					G1790A	HB	32	107	32	0	0	107	0	0	0.99
			Caucasian	Ovarian	C1772T	HB	49	107	34	14	1	68	37	2	0.23
					G1790A	HB	49	107	47	2	0	107	0	0	0.99
			Caucasian	Endometrial	C1772T	HB	21	107	4	12	5	68	37	2	0.23
					G1790A	HB	21	107	21	0	0	107	0	0	0.99
Li	2007	USA	mixed	Prostate	C1772T	PB	1041	1234	818	209	14	995	221	18	0.16
					G1790A	PB	1066	1264	1053	13	0	1247	17	0	0.81
Orr-Urtreger	2007	Israel	Caucasian	Prostate	C1772T	PB	402	300	287	99	16	217	80	3	0.14
					G1790A	PB	200	300	198	2	0	298	2	0	0.95
Apaydin	2008	Turkey	Caucasian	Breast	C1772T	PB	102	102	79	21	2	68	29	5	0.42
					G1790A	PB	102	102	102	0	0	98	4	0	0.84
Lee	2008	Korea	Asian	Breast	C1772T	PB	1332	1369	1207	119	6	1245	123	1	0.25
Kim	2008	Korea	Asian	Breast	C1772T	HB	90	102	81	8	1	93	9	0	0.64
					G1790A	HB	90	102	87	3	0	94	7	1	0.06
Nadaoka	2008	Japan	Asian	Bladder	C1772T	HB	219	461	197	21	1	419	42	0	0.35
					G1790A	HB	219	461	204	13	2	421	40^*^	-	0.46
Jacobs	2008	USA	mixed	Prostate	C1772T	HB	1420	1450	1156	252	12	1138	284	28	0.04
Horree	2008	Netherland	Caucasian	Endometrial	C1772T	PB	58	559	50	5	3	463	84	12	0.01
Naidu	2009	Malaysia	Asian	Breast	C1772T	PB	410	275	294	100	16	222	50	3	0.92
					G1790A	PB	410	275	332	72	6	232	41	2	0.90
Chen	2009	Taiwan	Asian	Oral	C1772T	PB	174	347	163	10	1	334	13	0	0.72
					G1790A	PB	174	347	153	20	1	333	14	0	0.70
Konac	2009	Turkey	Caucasian	Lung	C1772T	PB	141	156	110	31	0	111	43	2	0.34
					G1790A	PB	141	156	141	1	0	154	2	0	0.94
Morris	2009	UK	Caucasian	Renal	C1772T	PB	332	313	290	39	3	262	46	5	0.08
					G1790A	PB	325	309	313	10	2	294	15	0	0.66
Foley	2009	Ireland	Caucasian	Prostate	C1772T	PB	95	188	65	30	0	175	13	0	0.62
Li	2009	China	Asian	Gastric	C1772T	HB	87	106	83	4	0	93	13	0	0.50
					G1790A	HB	87	106	74	13	0	100	6	0	0.76
Munoz-															
Guerra	2009	Spain	Caucasian	Oral	C1772T	PB	70	148	57	6	7	113	27	8	<0.01
					G1790A	PB	64	139	40	21	3	130	9	0	0.69
Kim	2010	Korea	Asian	Cervical	C1772T	HB	199	214	177	22	0	187	27	0	0.32
					G1790A	HB	199	213	187	12	0	200	12	1	0.10
Shieh	2010	Taiwan	Asian	Oral	C1772T	HB	305	96	282	23	0	89	7	0	0.71
					G1790A	HB	305	96	281	24	0	89	7	0	0.71
Knechtal	2010	Austria	Caucasian	Colorectal	C1772T	PB	368	2156	291	77^**^	-	1773	383^*^	-	>0.05
					G1790A	PB	367	2156	356	11^*^	-	2080	76^*^	-	>0.05
Hsiao	2010	Taiwan	Asian	Hepatocellul-ar	C1772T	HB	102	347	94	8	0	334	13	0	0.72
					G1790A	HB	102	347	87	15	0	333	14	0	0.70
Xu	2011	China	Asian	Glioma	C1772T	HB	150	150	121	27	2	135	14	1	0.35
Putra	2011	Japan	Asian	Lung	C1772T	PB	83	110	74	9	0	98	12	0	0.55
					G1790A	PB	83	110	72	9	2	101	9	0	0.65
Kang	2011	Korea	Asian	Colorectal	C1772T	PB	50	50	46	4^**^	-	38	12^**^	-	<0.01
Wang	2011	China	Asian	Pancreatic	C1772T	HB	263	271	209	54	0	242	29	0	0.35
					G1790A	HB	263	271	198	65	0	249	22	0	0.49
Zagouri	2012	Greece	Caucasian	Breast	C1772T	HB	113	124	98	15	0	107	17	0	0.41
Kuo	2012	Taiwan	Asian	Lung	C1772T	HB	285	300	153	94	38	216	73	11	0.13
					G1790A	HB	285	300	150	94	41	215	74	11	0.15
Qin	2012	China	Asian	Renal	C1772T	HB	620	623	572	46	2	578	43	2	0.22
					G1790A	HB	620	623	575	45	0	584	39	0	0.42
Li	2012	China	Asian	Prostate	C1772T	HB	662	716	612	48	2	659	57	0	0.27
					G1790A	HB	662	716	614	47	1	685	31	0	0.55
Alves	2012	Brazil	mixed	Oral	C1772T	PB	40	88	0	1	39	0	85	3	<0.01
					G1790A	PB	40	88	2	1	37	81	7	0	0.70
Ruiz-Tovar	2012	Spain	Caucasian	Pancreatic	C1772T	PB	59	152	47	1	11	116	28	8	0.002
					G1790A	PB	59	152	54	2	3	142	10	0	0.68
Fu	2013	China	Asian	Cervical	C1772T	HB	518	553	467	49	2	492	60	1	0.55
					G1790A	HB	509	553	489	20	0	510	42	1	0.89
Ribeiro	2013	Portugal	Caucasian	Breast	C1772T	PB	96	74	74	21	1	61	7	4	0.001
					G1790A	PB	96	74	96	0	0	74	0	0	0.99
Mera-															
Menendez	2013	Spain	Caucasian	Glottic											
larynx	C1772T	HB	118	148	85	18	15	113	27	8	0.001				
					G1790A	HB	111	139	107	4	0	130	9	0	0.69
Total					C1772T		10869	14289	8994	1568	307	12181	1897	211	
					G1790A		7117	10442	6416	589	112	9922	494	26	

W: wide type alleles (1772C or 1790G); M: mutant type alleles (1772 T or 1790A); HWE: Hardy-Weinberg Equilibrium; PB: population based; HB: hospital based.

Mixed: Caucasian and African-American; HNSCC: head and neck squamous cell carcinoma.

*Frequency of genotypes “AA + AG”; **Frequency of genotypes “TT + TC”.

### Quantitative data synthesis

**Figure 2:**
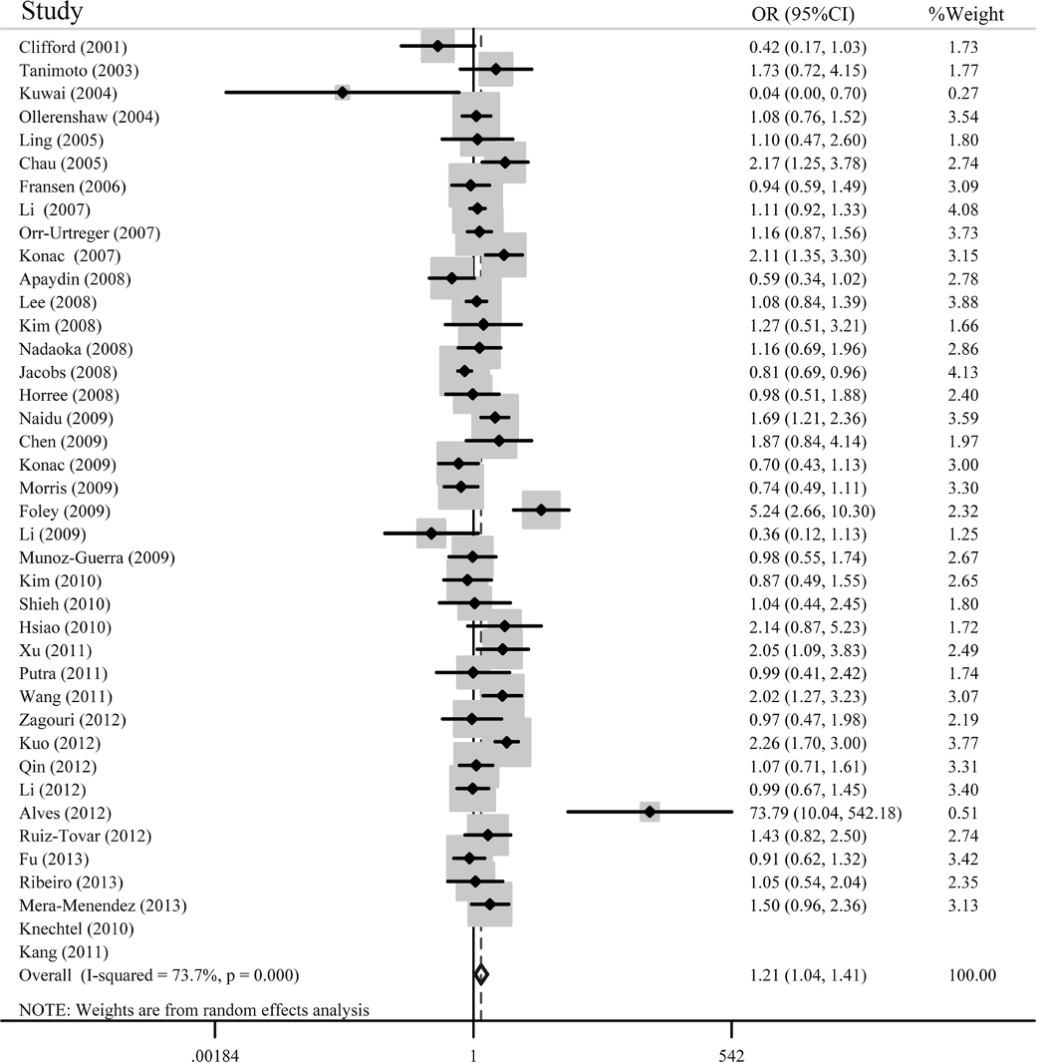
**Forest plot of the association between cancer risk and the HIF-1α C1772T polymorphism using the allelicmodel (T vs. C).**

For the HIF-1α C1772T polymorphism, the overall results from the eligible studies demonstrated a significant association between the HIF-1α C1772T polymorphism and an increased cancer risk in four genetic models (TT *vs.* CC: OR = 1.63, 95% CI = 1.02-2.60; CT + TT *vs.* CC: OR = 1.15, 95% CI = 1.01-1.34; TT *vs.* CT + CC: OR = 2.11, 95% CI = 1.32-3.77; T *vs.* C: OR = 1.21, 95% CI = 1.04-1.41). In the subgroup analysis by cancer type, the HIF-1α C1772T polymorphism significantly increased the risk of breast cancer in Asians (TT *vs.* CC: OR = 4.42, 95% CI = 1.60-12.21; TT *vs.* CT + CC: OR = 4.16, 95% CI = 1.51-11.48; T *vs.* C: OR = 1.28, 95% CI = 1.05-1.55), other cancers (TT *vs.*CC: OR = 3.18, 95% CI = 1.90-5.32; TT *vs.* CT + CC: OR = 3.31, 95% CI = 1.98-5.53; T *vs.* C: OR = 1.47, 95% CI = 1.10-1.96) and lung cancer (TT *vs.* CT + CC: OR = 3.27, 95% CI = 1.73-6.17 ). When the data was stratified by ethnicity, the HIF-1α C1772T polymorphism was significantly correlated with an increased cancer risk in Asian population (TT *vs*. CC: OR = 4.10, 95% CI = 2.49-6.76; CT + TT *vs.* CC: OR = 1.29, 95% CI = 1.04-1.58; TT *vs.* CT + CC: OR = 3.67, 95% CI = 2.23-6.02; T *vs.* C: OR = 1.28, 95% CI = 1.04-1.57) and Caucasian population (TT *vs.* CT + CC: OR = 1.95, 95% CI = 1.14-3.31). In the analysis stratified by the sources of controls, a significant association was observed in the hospital-based group (CT + TT *vs.* CC: OR = 1.28, 95% CI = 1.01-1.62; T *vs.* C: OR = 1.33, 95% CI = 1.04-1.71) and the population-based group (TT *vs.* CT + CC: OR = 2.01, 95% CI = 1.10-3.71). Sensitivity analyses were carried out to assess the stability of the results by conducting analyses of studies with controls in HWE. The results showed significantly increased cancer risk (TT *vs.* CC: OR = 2.47, 95% CI = 1.81-3.36; CT + TT *vs.* CC: OR = 1.25, 95% CI = 1.05-1.49; TT *vs*. CT + CC: OR = 2.43, 95% CI = 1.41-4.19; T *vs.* C: OR = 1.27, 95% CI = 1.06-1.52). The other results for the HIF-1α C1772T polymorphism were similar to those when the studies with controls not in HWE were included. The main results of this pooled analysis are shown in Table 2. Figure 2 shows the forest plot of the association between cancer risk and the HIF-1α C1772T polymorphism under the allelic model.

**Table 2:** **Meta-analysis of the HIF-1α C1772T polymorphism and cancer risk**

Variables			TT ***vs.***CC				CT ***vs.***CC				CT + TT ***vs.***CC				TT ***vs.***CT + CC				T ***vs.***C		
	***Study***	***Case/control***	***I*** ^***2***^	***Phet***	***OR (95% CI)***	***Case/control***	***I*** ^***2***^	***Phet***	***OR (95% CI)***	***Case/control***	***I*** ^***2***^	***Phet***	***OR (95% CI)***	***case/control***	***I*** ^***2***^	***Phet***	***OR (95% CI)***	***Case/control***	***I*** ^***2***^	***Phet***	***OR (95% CI)***
Overall	40	9301/12392	67	<0.001	1.63 (1.02-2.60)^*^	10562/14078	68	<0.001	1.08 (0.92-1.26)^*^	10958/14676	70	<0.001	1.15 (1.01-1.34)^*^	10540/12470	71	<0.001	2.11 (1.32-3.37)^*^	21738/28578	76	<0.001	1.21 (1.04-1.41)^*^
Overall in HWE	31	7429/9947	59	0.02	2.21 (1.27-3.83)^*^	8481/11109	64	<0.001	1.15 (0.98-1.36)^*^	8604/11556	70	<0.001	1.20 (1.02-1.41)^*^	8275/9350	49	0.01	2.13 (1.28-3.55)^*^	17208/22338	76	<0.001	1.22 (1.03-1.44)^*^
Cancer type																					
Cervical	3	664/750	66	0.09	10.11 (2.55-40.05)	739/871	60	0.08	0.98 (0.72-1.34)	749/874	80	0.01	1.32 (0.61-2.87)^*^	749/874	51	0.15	8.55 (2.28-32.13)	2369/1748	88	<0.001	1.41 (0.59-3.35)^*^
Breast	6	1859/1809	62	0.03	1.41 (0.34-5.75)^*^	2117/2033	37	0.16	1.01 (0.91-1.33)	2143/2046	46	0.1	1.13 (0.94-1.36)	2143/2046	60	0.04	1.38 (0.35-5.46)^*^	4286/4092	56	0.04	1.09 (0.80-1.48)^*^
Breast in HWE	5	1784/1744	55	0.08	2.30 (1.08-4.91)	2022/1963	35	0.19	1.07 (0.88-1.29)	2047/1972	56	0.06	1.12 (0.92-1.35)	2047/1972	49	0.12	2.27 (1.06-4.82)	4154/3944	65	0.02	1.09 (0.76-1.55)^*^
Breast in Asian	3	1605/1564	0	0.93	4.42 (1.60-12.21)	1809/1742	36	0.21	1.14 (0.92-1.41)	1832/1746	51	0.13	1.22 (0.99-1.49)	1832/1746	0	0.91	4.16 (1.51-11.48)	3664/3492	55	0.11	1.28 (1.05-1.55)
Lung	3	375/438	75	0.04	1.41 (0.07-30.44)^*^	471/553	75	0.02	1.13 (0.59-2.19)^*^	509/566	86	0.01	1.19 (0.51-2.76)^*^	509/566	71	0.07	3.27 (1.73-6.17)	1018/1132	89	<0.001	1.19 (0.50-2.86)^*^
Colorectal	4	599/2123	-	-	-	624/2175	79	0.03	0.24 (0.01-5.51)^*^	627/2177	71	0.02	1.12 (0.57-2.18)^*^	627/2177	-	-	-	1254/4354	80	0.02	0.26 (0.01-6.38)^*^
Prostate	6	3149/3415	70	0.01	1.34 (0.54-3.31)^*^	3766/4032	86	<0.001	1.34 (0.93-1.92)^*^	3816/4084	87	<0.001	1.36 (0.95-1.96)^*^	3816/4084	69	0.01	1.31 (0.54-3.20)^*^	7632/8168	87	<0.001	1.35 (0.96-1.89)^*^
Prostate in HWE	4	1814/2067	59	0.09	1.57 (0.89-2.75)	2168/2417	88	<0.001	1.42 (0.84-2.40)^*^	2200/2438	87	0.01	1.50 (0.89-2.40)^*^	2200/2438	61	0.08	1.55 (0.89-2.72)	4400/4876	85	<0.001	1.44 (0.93-2.21)^*^
Renal	4	1015/1035	25	0.26	0.28 (0.12-1.28)	1065/1157	74	0.01	0.62 (0.31-1.24)^*^	1160/1241	70	0.02	0.62 (0.33-1.18)^*^	1160/1241	21	0.29	1.37 (0.92-2.04)	2320/2482	44	0.15	0.91 (0.73-1.12)
Renal in HWE	2	867/847	0	0.62	0.67 (0.21-2.13)	947/929	13	0.28	0.92 (0.67-1.26)	952/936	29	0.24	0.90 (0.67-1.22)	952/936	0	0.64	0.69 (0.22-2.17)	1904/1872	37	0.21	0.89 (0.67-1.19)
Oral	4	549/547	0	0.46	2.01 (0.75-5.41)	542/668	50	0.14	0.90 (0.55-1.47)	589/679	16	0.3	1.04 (0.66-1.64)	589/679	93	<0.001	22.82 (0.28-1887.72)^*^	1178/1358	88	<0.001	2.52 (0.71-8.98)^*^
Oral in HWE	2	446/423	-	-	-	478/443	0	0.5	1.28 (0.69-2.38)	479/443	0	0.4	1.35 (0.73-2.49)	479/443	-	-	-	958/886	0	0.32	1.41 (0.78-2.56)
Other	12	1033/2151	30	0.2	3.18 (1.90-5.32)	1190/2445	67	<0.001	1.18 (0.79-1.78)^*^	1276/2622	60	<0.001	1.34 (0.95-1.87)^*^	1276/2622	0	0.52	3.31 (1.98-5.53)	2434/4940	58	0.01	1.47 (1.10-1.96)^*^
Other in HWE	9	880/1032	56	0.08	5.10 (1.72-15.07)	1032/1758	60	0.01	1.47 (0.97-2.21)^*^	1041/1763	64	0.01	1.52 (0.99-2.34)^*^	1041/1763	24	0.27	4.47 (1.53-13.00)	2082/3526	67	0.01	1.52 (1.02-2.28)^*^
Ethnicity																					
Asian	20	5124/5781	0	0.96	4.10 (2.49-6.76)	5678/6335	50	0.01	1.20 (0.99-1.46)^*^	5787/6400	75	<0.001	1.29 (1.04-1.58)^*^	5787/6400	0	0.98	3.67 (2.23-6.02)	11574/12800	61	<0.001	1.28 (1.04-1.57)
Caucasian	16	1791/4247	74	<0.001	1.54 (0.72-3.27)^*^	2220/4781	76	<0.001	0.93 (0.65-1.33)^*^	2385/4921	59	0.01	1.07 (0.80-1.43)^*^	2385/4921	58	0.003	1.95 (1.14-3.31)^*^	4770/9842	78	<0.001	1.20 (0.91-1.57)
Caucasian in HWE	9	1473/3153	76	<0.001	2.28 (0.62-8.35)^*^	1738/3152	79	<0.001	1.20 (0.99-1.46)^*^	1776/3535	82	<0.001	1.28 (0.88-1.86)^*^	1776/3535	69	0.002	2.08 (0.68-6.37)^*^	3552/7070	86	<0.001	1.34 (0.86-2.07)
Source of control																					
HB	17	4608/5249	77	<0.001	3.28 (1.29-8.30)^*^	5259/6029	60	<0.001	1.18 (0.96-1.45)^*^	5348/6086	72	<0.001	1.28 (1.01-1.62)^*^	5348/6086	71	<0.001	2.85 (1.24-6.54)^*^	10696/12172	80	<0.001	1.33 (1.04-1.71)^*^
HB in HWE	15	3340/3962	35	0.13	4.88 (2.96-8.04)	3748/4467	56	0.01	1.24 (0.99-1.57)^*^	3810/4488	67	<0.001	1.33 (1.02-1.74)^*^	3810/4488	4	0.4	4.23 (2.58-6.93)	7620/8976	74	<0.001	1.38 (1.06-1.80)^*^
PB	23	4693/5303	54	0.01	1.33 (0.76-2.31)^*^	5303/7143	74	<0.001	0.99 (0.77-1.29)^*^	5521/8203	70	<0.001	1.10 (0.89-1.36)^*^	5521/8203	72	<0.001	2.02 (1.10-3.71)^*^	11042/16406	74	<0.001	1.18 (0.95-1.45)^*^
PB in HWE	15	4089/5985	49	0.04	1.51 (0.74-3.11)^*^	4733/6642	72	<0.001	1.10 (0.85-1.43)^*^	4794/6681	72	<0.001	1.17 (0.93-1.48)^*^	4794/6681	46	0.63	1.51 (1.01-2.27)	9588/13362	75	<0.001	1.14 (0.89-1.45)^*^

HWE: Hardy-Weinberg Equilibrium; PB: population based; HB: hospital based; Phet: P value for heterogeneity. ^*^Random-effects model was used when P value for heterogeneity test <0.05; otherwise, fixed-effects model was used.

**Figure 3:**
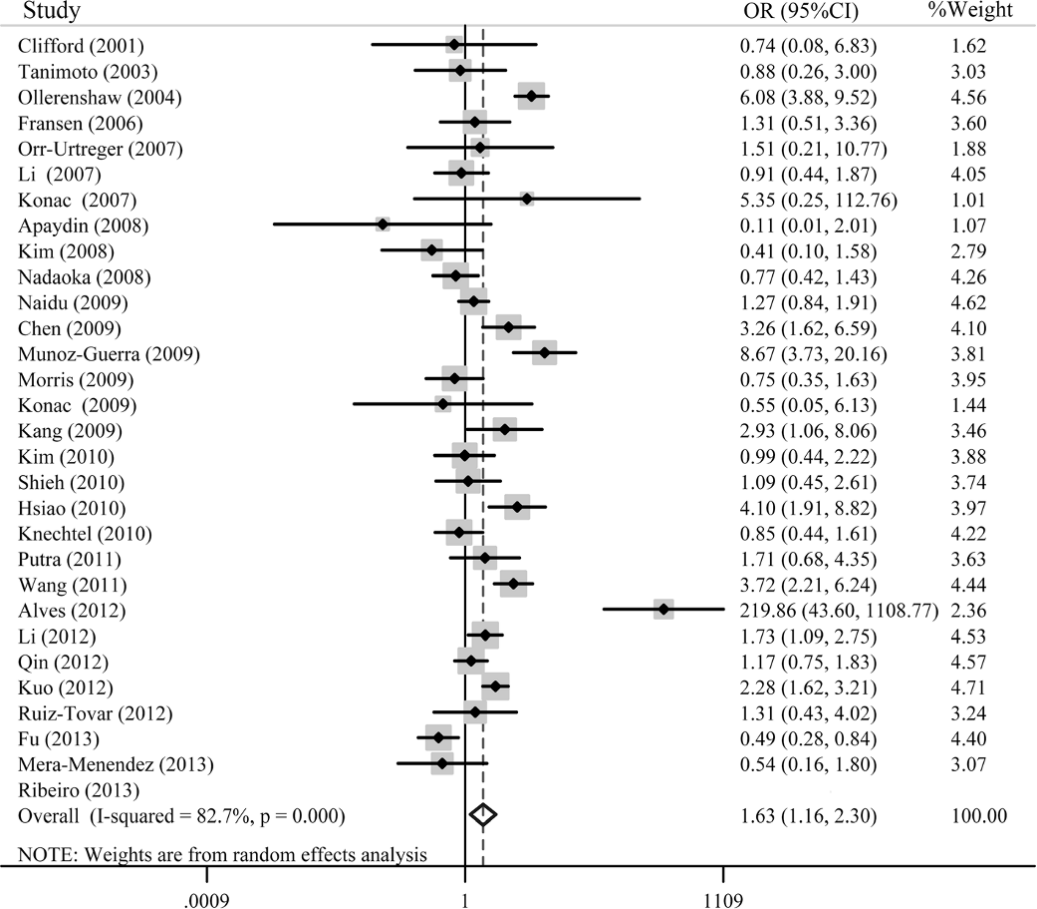
**Overall association between the HIF-1α G1790A polymorphism and cancer risk for all subjects using the dominant model (GA + AA vs. GG).**

For HIF-1α G1790A polymorphism, as shown in Table 3, the association between the HIF-1α G1790A polymorphism and increased cancer risk was significant for the pooled ORs under all of the genetic models (AA *vs*. GG: OR = 5.11, 95% CI = 2.08-12.56; GA *vs.* GG: OR = 1.45, 95% CI = 1.05-1.99; AA + AG *vs.* GG: OR = 1.63, 95% CI = 1.16-2.30; AA *vs.* GA + GG: OR = 4.41, 95% CI = 1.80-10.84; A *vs.* G: OR = 1.77, 95% CI = 1.23-2.25). In the subgroup analysis by cancer type, a significant association was observed in lung cancer (AA *vs.* GG: OR = 5.42, 95% CI = 2.74-10.70; GA *vs.* GG: OR = 1.72, 95% CI = 1.22-2.41; AA + AG *vs.* GG: OR = 2.14, 95% CI = 1.56-2.94; AA *vs.* GA + GG: OR = 4.52, 95% CI = 2.31-8.83; A *vs.* G: OR = 2.27, 95% CI = 1.74-2.95), pancreatic cancer (AA + AG *vs.* GG: OR = 3.14, 95% CI = 1.99-2.97; A *vs*. G: OR = 3.08, 95% CI = 1.98-4.78) and renal cancer (AA *vs.* GA + GG: OR = 3.09, 95% CI = 1.38-6.92). When the data were stratified by ethnicity, significantly increased cancer risk was observed in Asian population and Caucasian population. When the studies were stratified by the source of controls, a significant association was observed for population-based controls under the homozygote model, the dominant comparison model and the allelic model. Sensitivity analyses were conducted after the removal of the studies with controls not in HWE, the results for the HIF-1α G1790A polymorphism were similar to those when the studies with controls not in HWE were included. Table 3 shows the main results of this pooled analysis for the HIF-1α G1790A polymorphism. Figure 3 shows the forest plot of the association between cancer risk and the HIF-1α G1790A polymorphism under the dominant model.

**Table 3:** **Meta-analysis of the HIF-1α G1790A polymorphism and cancer risk**

Variables			AA ***vs.***GG				GA ***vs***. GG				AA + AG ***vs.***GG				AA ***vs***.GA + GG				A ***vs.***G		
	***Study***	***Case/control***	***I*** ^***2***^	***Phet***	***OR (95% CI)***	***Case/control***	***I*** ^***2***^	***Phet***	***OR (95% CI)***	***Case/control***	***I*** ^***2***^	***Phet***	***OR (95% CI)***	***Case/control***	***I*** ^***2***^	***Phet***	***OR (95% CI)***	***Case/control***	***I*** ^***2***^	***Phet***	***OR (95% CI)***
Overall	30	6538/9948	57	0.01	5.11 (2.08-12.56)^*^	7005/10442	77	<0.001	1.45 (1.05-1.99)^*^	7117/10442	83	<0.001	1.63 (1.16-2.30)^*^	7117/10442	58	0.01	4.41 (1.80-10.84)^*^	14234/20884	86	<0.001	1.77 (1.23-2.25)^*^
Overall in HWE	29	6449/9699	61	0.003	5.14 (1.67-15.86)^*^	6873/10138	69	<0.001	1.35 (1.01-1.81)^*^	6971/10154	79	<0.001	1.53 (1.10-2.12)^*^	6971/10154	60	0.004	4.80 (1.58-14.55)^*^	13942/20308	85	<0.001	1.70 (1.17-2.46)^*^
Cancer type																					
Breast	4	623/501	0	0.34	1.44 (0.38-5.44)	692/550	53	0.12	1.03 (0.70-1.52)	698/553	60	0.08	1.05 (0.72-1.53)	698/553	0	0.36	1.41 (0.37-5.40)	1396/1466	65	0.56	1.07 (0.76-1.52)
Cervical	3	708/819	0	0.99	0.35 (0.04-3.39)	740/871	57	0.13	0.62 (0.40-0.98)	740/837	51	0.15	0.60 (0.38-0.94)	740/837	0	0.99	0.36 (0.04-3.450	1480/1746	42	0.19	0.59 (0.38-0.91)
Oral	4	517/633	75	0.02	72.11 (2.08-2502.44)^*^	542/670	70	0.02	3.17 (1.26-7.92)^*^	583/670	92	<0.001	7.92 (1.58-39.64)^*^	583/670	75	0.02	58.05 (1.70-1985.77)^*^	1166/1340	96	0.01	9.66 (1.31-71.15)^*^
Prostate	3	1866/2230	-	-	-	1927/2280	1	0.37	1.42 (0.97-2.07)	1928/2280	7	0.34	1.44 (0.98-2.10)	1928/2280	-	-	-	3856/4560	10	0.33	1.45 (0.99-2.11)
Renal	4	1016/1267	0	0.95	5.10 (2.21-11.73)	1123/1354	92	<0.001	1.51 (0.45-5.05)^*^	1139/1364	92	<0.001	1.58 (0.49-5.04)^*^	1139/1364	0	0.76	3.09 (1.38-6.92)	2278/2728	89	<0.001	1.53 (0.60-3.92)^*^
Renal in HWE	3	937/1018	-	-	-	991/1076	0	0.42	1.00 (0.69-1.47)	993/1076	0	0.6	1.04 (0.71-1.52)	993/1076	-	-	-	1986/2152	0	0.78	1.07 (0.74-1.55)
Lung	3	405/481	0	0.87	5.42 (2.74-10.70)	466/555	0	0.57	1.72 (1.22-2.41)	509/566	0	0.46	2.14 (1.56-2.94)	509/566	0	0.79	4.52 (2.31-8.83)	1018/1132	0	0.48	2.27 (1.74-2.95)
Colorectal	2	545/2327	-	-	-	554/2336	-	-	-	554/2336	0	0.45	0.97 (0.57-1.63)	554/2336	-	-	-	1108/4672	-	-	-
Pancreatic	2	255/391	-	-	-	319/423	82	0.02	1.61 (0.24-10.76)^*^	322/423	63	0.1	3.14 (1.99-4.97)	322/423	-	-	-	644/846	0	0.42	3.08 (1.98-4.78)
Other	7	593/1377	-	-	-	642/1377	74	<0.001	1.53 (0.65-3.59)^*^	644/1377	72	<0.001	1.57 (0.70-3.53)^*^	644/1377	-	-	-	1288/2754	69	0.01	1.57 (0.75-3.30)^*^
Ethnicity																					
Asian	15	3607/4263	13	0.33	3.50 (2.05-5.98)	4010/4614	74	<0.001	1.44 (1.04-1.99)^*^	4063/4630	76	<0.001	1.49 (1.07-2.08)^*^	4063/4630	0	0.45	3.12 (1.83-5.32)	8126/9260	77	<0.001	1.49 (1.08-2.05)^*^
Caucasian	13	1829/4357	0	0.69	6.63 (3.11-14.12)	1926/4450	81	<0.001	1.36 (0.58-3.19)^*^	1948/4460	82	<0.001	1.45 (0.69-3.04)^*^	1948/4460	0	0.49	4.21 (2.04-8.71)	3896/8920	75	<0.001	1.65 (0.84-3.24)^*^
Caucasian in HWE	12	1750/4108	0	0.74	12.40 (2.19-70.22)	1794/4172	68	0.01	1.10 (0.48-2.49)^*^	1802/4172	67	0.01	1.22 (0.62-2.37)^*^	1802/4172	0	0.79	11.37 (2.02-63.93)	3604/8344	68	0.01	1.65 (1.17-2.32)^*^
Source of control																					
HB	13	3197/3945	45	0.12	1.54 (0.35-6.70)	3510/4234	77	<0.001	1.37 (0.92-2.05)^*^	3554/4248	79	<0.001	1.40 (0.93-2.11)^*^	3554/4248	35	0.19	3.13 (1.74-5.62)	7108/8496	79	<0.001	1.38 (0.93-2.05)^*^
PB	17	3133/5705	66	0.01	11.55 (6.62-20.12)^*^	3295/5882	78	<0.001	1.51 (0.88-2.58)^*^	3563/6194	85	<0.001	1.90 (1.06-3.39)^*^	3563/6194	69	0.002	10.27 (2.42-43.63)^*^	6726/11788	89	<0.001	2.25 (1.18-4.29)^*^
PB in HWE	16	3054/5456	67	0.006	15.51 (2.53-94.94)^*^	3163/5604	60	0.01	1.34 (0.85-2.11)^*^	3417/5906	81	<0.001	1.71 (0.97-3.03)^*^	3417/5906	66	0.007	14.20 (2.38-84.61)^*^	6434/11212	89	<0.001	2.33 (1.91-2.84)^*^

HWE: Hardy-Weinberg Equilibrium; PB: population based; HB: hospital based; Phet: P value for heterogeneity. ^*^Random-effects model was used when P value for heterogeneity test <0.05; otherwise, fixed-effects model was used.

### Test of heterogeneity

There was significant heterogeneity observed in the allelic comparison model, the dominant comparison model and the heterozygote comparison model (Tables 2 and 3), and the heterogeneity was effectively decreased or removed in the subgroups stratified by ethnicity, cancer types and source of controls (Tables 2 and 3).

### Sensitivity analysis

**Figure 4:**
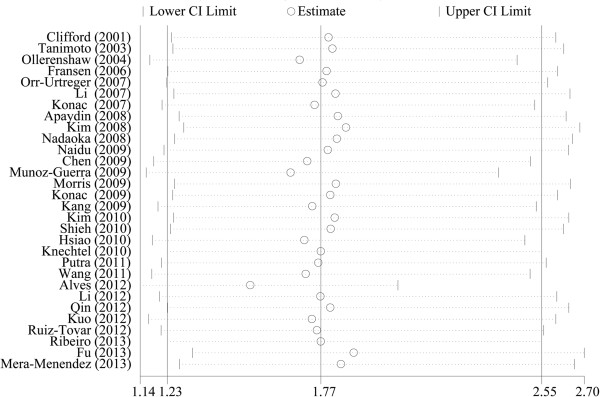
**The influence of individual studies on the summary odds ratio (OR) for the HIF-1α G1790A polymorphism.**

We performed sensitivity analysis by removing each individual study (including the restudies with controls not in HWE) sequentially for both the HIF-1α C1772T and the HIF-1α G1790A polymorphism (Figure 4 and Additional file 1). The results indicated that the overall significance of the pooled ORs was not altered by any single study in the genetic models for the HIF-1α C1772T/G1790A polymorphisms and cancer susceptibility, suggesting stability and reliability in our overall results.

### Bias diagnostics

**Figure 5:**
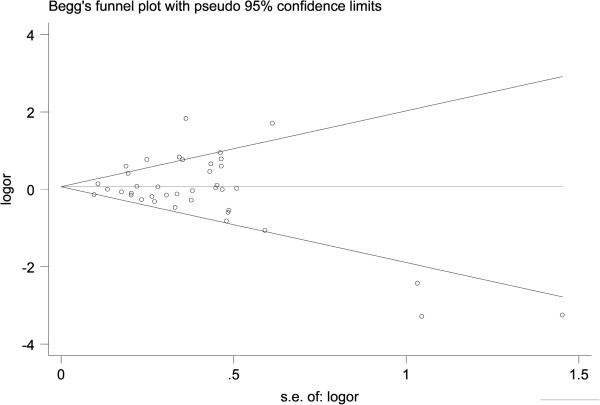
**Begg’s funnel plot for evaluating the publication bias of the meta-analysis for the HIF-1α C1772T polymorphism.**

**Figure 6:**
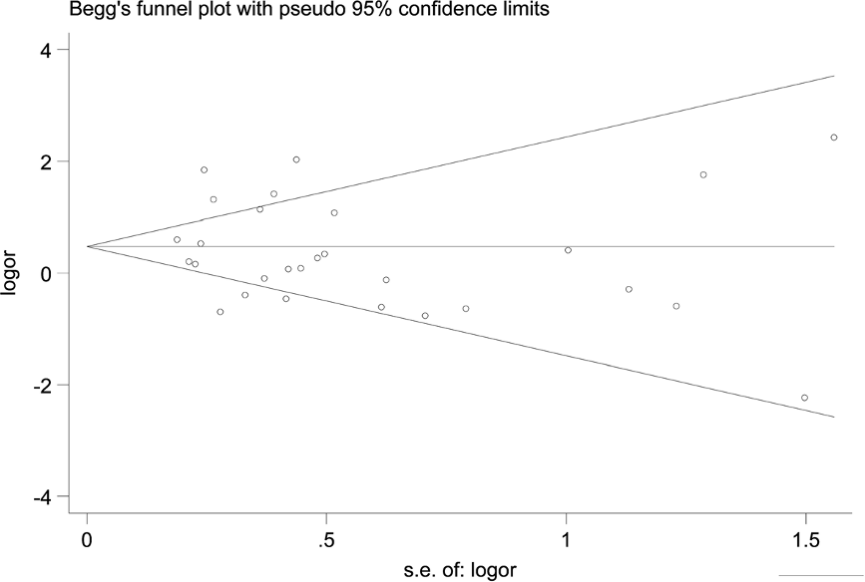
**Begg’s funnel plot for evaluating the publication bias of the meta-analysis for the HIF-1α G1790A polymorphism.**

A Begg’s funnel plot and Egger’s test were used to assess the publication bias in this meta-analysis. As shown in Figure 5, for the HIF-1α C1772T polymorphism, the funnel plots for the comparison of the five models appear to be basically symmetric. The Egger’s linear regression test did not show any evidence of significant publication bias in five models (TT *vs.* CC: t = 0.50, *P* = 0.62; TC *vs.* CC: t = -0.19, *P* = 0.85; TT *vs.* CT + CC: t = 1.11, *P* = 0.28; T *vs.* C: t = 1.39, *P* = 0.17; CT + TT *vs*. CC: t = 0.59, *P* = 0.56). For the HIF-1α G1790A polymorphism, no visual publication bias was detected in the funnel plot (Figure 6) and the result showed no significant evidence of a publication bias in five models(AA *vs.* GG: t = 0.03, *P* = 0.98; GA *vs.* GG: t = -0.86, *P* = 0.40; AA *vs.* GA + GG: t = 0.33, *P* = 0.75; AA + AG *vs.*GG: t = -0.40, *P* = 0.69; A *vs.* G: t = -0.41, *P* = 0.68).

## Discussion

HIF-1 is a heterodimeric transcription factor and a key regulator of the cellular response to hypoxia [5]. It is composed of HIF-1α and HIF-1β subunits, which are members of the bHLH-PAS transcription factor family. HIF-1α is a unique O_2_-regulated subunit that determines the function of HIF-1. HIF-1α upregulates the expression of genes whose protein products function to increase O_2_ availability or to allow metabolic adaptation to O_*2*_ deprivation, including VEGF, Epo, NOS2 and others. Most of these aforementioned proteins have been implicated in tumor development and progression [35, 64, 65]. Recent studies have reported that the overexpression of HIF-1α is significantly associated with cell proliferation, tumor susceptibility, tumor size, lymph node metastasis and prognosis [12, 35, 66]. The HIF-1α gene is located on chromosome 14q21-24 and contains a total of 35 common SNPs, according to the dbSNP database (http://www.ncbi.nlm.nih.gov/SNP). Two polymorphisms, C1772T (rs11549465) and G1790A (rs11549467), result in an amino acid substitution of proline to serine and alanine to threonine, respectively, and the present studies show that C1772T (rs11549465) is not in substantial linkage disequilibrium (LD) with G1790A (rs11549467) (R^2^ = 0.002). Under normoxic condition, the hydroxylation of proline 402 and proline 564 occurs within the oxygen-dependent degradation (ODD) domain of HIF-1α, and HIF-1α is rapidly degraded. The two SNPs examined here are located within the ODD/pVHL binding domain in exon 12 of the HIF-1α gene and may enhance the transcription activity of the HIF-1α gene by causing structural changes, increasing the stability of HIF-1α protein and affecting the expression of downstream target genes [8, 14, 17]. Over the last few years, a great number of studies have been performed to investigate the association between these HIF-1α polymorphisms and cancer risk in different populations. However, the results of these studies remain inconclusive. In a meta-analysis conducted by Zhao *et al.* in 2009 [67], the HIF-1 C1772T polymorphism was reported to be associated with increased cancer risk, while no significant association was found between the HIF-1α G1790A polymorphism and cancer risk. Additionally, Li *et al.* reported that the HIF-1α C1772T polymorphism correlates with urinary cancer risk in Caucasian population, and the G1790A polymorphism may increase the risk of prostate cancer [68]. Due to the important role of HIF-1α polymorphisms in the development of cancer and due to the limited statistical power of the previous studies, we conducted a comprehensive literature search and performed a meta-analysis on all of the available case-control studies to systematically evaluate the exact relationship between the C1772T/G1790A polymorphisms in HIF-1α and cancer susceptibility.

Regarding the HIF-1α C1772T polymorphism, our results suggested a significant association in four genetic comparison models, providing convincing evidence that the HIF-1α C1772T polymorphism may be a risk factor in cancer development. When sensitivity analyses were performed, the results were similar to those when the studies with controls not in HWE were included, suggesting that our results were very robust. Moreover, when the data were stratified by cancer type, a significant association was observed between the C1772T polymorphism and breast cancer in Asians. This may be due to the specific genetic variant induced over-expression of HIF-1 under hypoxic condition in breast cancer cells, and the different life style, ethnicity and body composition between Asians and Caucasians, which could contribute to the results. A significant association was also observed in lung cancer. When subgroup analyses were performed according to ethnicity and source of controls, a significant association was found in Asian population, Caucasian population and in hospital-based studies. Zhao *et al.* [67] reported that the genotype TT was significantly associated with an increased cancer risk in Asians, but the CI was very wide due to the lack of mutant homozygotes in Asians. In our meta-analysis, we also found that the C1772T polymorphism was a risk factor in Asians (Dominant model: OR = 1.29, 95% CI = 1.04-1.58; Allelic model: OR = 1.47, 95% CI = 1.04-1.57). Beyond that, we had not found any significant associations in prostate cancer, renal cancer or oral cancer.

For the HIF-1α G1790A polymorphism, the pooled results from all of the eligible studies suggested that the G1790A polymorphism in HIF-1α is significantly associated with an increased cancer risk in all of the genetic models. We also conducted subgroup analyses based on the cancer type, ethnicities and source of controls. In the subgroup analysis according to cancer type, the results suggested that the HIF-1α G1790A polymorphism significantly increased the risk of lung cancer, renal cancer, oral cancer and pancreatic cancer, but the CI for the oral cancer subgroup was very wide. This may be due to the lack of mutant homozygotes detected, and the association could have been caused by chance. More studies based on large populations should be prusued. The study reported by Putra *et al.* indicated that even though they did not found any significant differences in genotype for G1790A between lung cancer patients and healthy controls, however, the G1790A variant allele was significantly higher in lung cancer patients, and TP53 LOH and 1p34 LOH were more frequently observed in individuals with the HIF-1α G1790A polymorphism, suggesting that this polymorphism may induce mutations in some tumor suppressor genes involved in lung cancer development [46]. Here, we found a significant association between the G1790A polymorphism and lung cancer risk. When the data were stratified according to ethnicity classification and source of controls, similar to the C1772T polymorphism, significantly increased risks were also found in Asian populations, Caucasian populations and population-based studies. After sensitivity analyses were performed, our results did not vary substantially, which strongly suggests an association between the HIF-1α G1790A polymorphism and increased cancer risk. One important factor that could influence the results is heterogeneity. In our study, significant heterogeneity existed in the analysis of the heterozygote model, the dominant model and the allelic model for the HIF-1α C1772T/G1790A polymorphism. When we performed a subgroup analysis according to cancer type, ethnicity or source of controls, the heterogeneity was reduced significantly or disappeared. The significant heterogeneity may due to the differences in ethnicity or cancer types or even in the selection of the controls. Furthermore, publication bias was not observed in our meta-analysis of the HIF-1α G1790A/C1772T polymorphisms. We also performed a sensitivity analysis to evaluate the sources of heterogeneity. The pooled ORs did not vary substantially, indicating that the results of our meta-analysis are robust and reliable.

To a certain extent, our meta-analysis still includes several limitations that should be interpreted and taken into consideration. First, in the era of GWAS, researchers can obtain the GWAS data for these two SNPs from all cancer studies and conduct a meta-analysis with the GWAS data instead of relying on published data, which may be biased toward positive findings. Second, the lack of observations concerning gene-gene and gene-environment interactions could influence our results. Third, although the total number of studies was not small, there were still not sufficient eligible studies for us to analyze different types of cancers, such as colorectal carcinoma, renal cell carcinoma or glioma; more studies are needed to explore the potential relationship between HIF-1αC1772T/G1790A polymorphisms and cancer susceptibility. Forth, the lack of detailed original data, such as the age and sex of the populations, smoking status, or alcohol consumption in the eligible studies may influence our extended analyses. However, our meta-analysis also has many advantages. First, we searched all possible publications, and the total number of eligible studies was much larger than other previously published meta-analyses; therefore, our results are more convincing. Second, no publication bias was detected in our meta-analysis. Finally, all of the data were extracted from well-selected studies, providing stronger statistical power for our study.

## Conclusions

In conclusion, this meta-analysis provides powerful evidence that both the C1772T and G1790A polymorphisms in the HIF-1α gene may contribute to individual susceptibility to cancers. It will be necessary to perform additional research to evaluate the relationship between HIF-1α C1772T/G1790A polymorphisms and cancer risk. Moreover, large sample case-control studies assessing gene-to-gene and gene-to-environment interactions are required to verify these findings.

## Authors’ information

Qing Yan, Pin Chen and Songtao Wang are joint first authors.

## Electronic supplementary material

Additional file 1: **The influence of individual studies on the summary odds ratio (OR) for the HIF-1α C1772T polymorphism.** (TIFF 271 KB)
