# Supplementary figures and images for: Association between HIF-1α C1772T/G1790A polymorphisms and cancer susceptibility: an updated systematic review and meta-analysis based on 40 case-control studies
Source: BMC Cancer. 2014 Dec 15;14:950. doi: 10.1186/1471-2407-14-950 (PMC4301938; doi:10.1186/1471-2407-14-950)

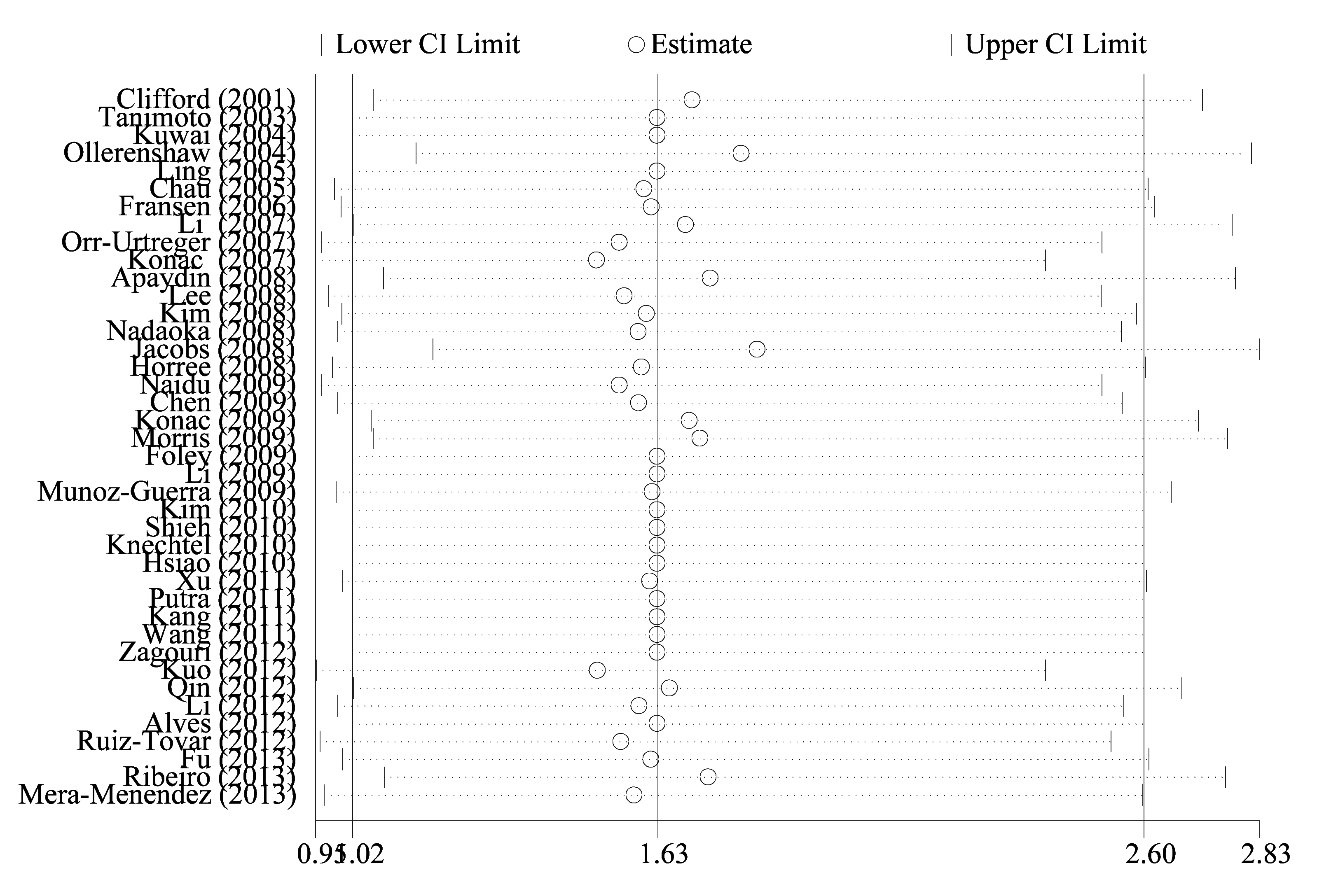

Supplement: Supplementary file 1 — Additional file 1: The influence of individual studies on the summary odds ratio (OR) for the HIF-1α C1772T polymorphism. (TIFF 271 KB) [file 12885_2013_5113_MOESM1_ESM.tiff]
